# First experience with a thermal-sprayed silver oxide-containing hydroxyapatite coating implant in two-stage total hip arthroplasty for the treatment of septic arthritis with hip osteoarthritis: A case report

**DOI:** 10.1016/j.ijscr.2020.11.032

**Published:** 2020-11-11

**Authors:** Akira Hashimoto, Motoki Sonohata, Masaru Kitajima, Shunsuke Kawano, Shuichi Eto, Masaaki Mawatari

**Affiliations:** a Department of Orthopaedic Surgery, Faculty of Medicine, Saga University, Nabeshima 5-1-1, Saga 849-8501, Japan; b Department of Orthopaedic Surgery, Shiroisi Kyouritu Hosptal, Fukuda 1296, Shiroishi-cho, Kishima-gun, Saga 849-1112, Japan

**Keywords:** Septic arthritis of the hip, Silver, Total hip arthroplasty

## Abstract

**Introduction:**

Septic arthritis of the hip joint in adults is a rare and potentially devasting disease. To the best of our knowledge, there have been no reports of two-stage total hip arthroplasty (THA) for the treatment of septic arthritis of the hip joint with a cementless hip implant that has antibacterial properties.

**Presentation of case:**

We present a case of two-stage THA with a thermal-sprayed silver oxide-containing hydroxyapatite coating (Ag-HA) implant to treat septic arthritis of the hip joint with hip osteoarthritis in an 80-year-old woman. There was no complication or recurrence at 28 months follow-up after 2nd-stage operation.

**Discussion:**

Ag-HA implants were found to have antibacterial activity within the subcutaneous tissues and bone, osteoconductive properties, and no adverse reactions *in vivo*. Moreover, no adverse events due to silver were reported in a clinical or radiographic study.

**Conclusion:**

To further reduce infection after two-staged THA for septic arthritis of the hip joint, antibacterial implants, such as an Ag-HA implant, may be used.

## Highlights

• Septic arthritis of the hip joint is relatively uncommon in adults.
• Ag-HA implants have not been used in two-stage total hip arthroplasty.
• Ag-HA implants may be used in two-staged total hip arthroplasty.

## 1 Introduction

Septic arthritis of the hip joint (SAHJ) in adults is a rare and potentially devasting disease [1]. The duration of symptoms is important for the selection of treatment [1]. Symptoms with an early onset can be treated with open or arthroscopic debridement [1]. Radical treatment is required for patients with prolonged symptoms or radiological destruction of the hip joint [1,2]. The conventional treatment for SAHJ is Girdlestone arthroplasty [3]. Currently, two-stage total hip arthroplasty (THA) with an interval antibiotic-loaded cement spacer is performed for the treatment of SAHJ [1,2]. However, there have been no reports of two-stage THA to treat SAHJ with a cementless hip implant that has antibacterial properties. Silver (Ag) is a well-known antibacterial agent with broad-spectrum activity, less bacterial resistance than that of antibiotics, and low toxicity toward humans [4]. In this case report, we report on a patient with SAHJ and hip osteoarthritis treated with two-stage THA using a thermal-sprayed silver oxide-containing hydroxyapatite coating (Ag-HA) implant, performed by an experienced orthopedic surgeon. This case was reported according to the SCARE criteria [5].

## 2 Case presentation

**Fig. 1:**
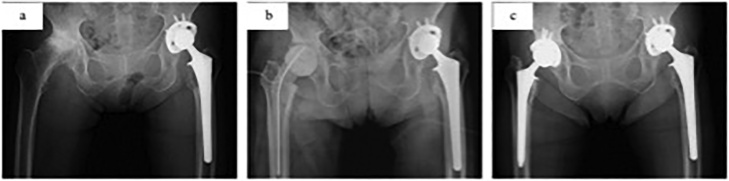
Anterior-posterior plain radiograph of the hip joint. (a) Plain radiograph before 1st-stage operation. (b) Plain radiograph after 1st-stage operation. (c) Plain radiograph at final follow-up.

**Fig. 2:**
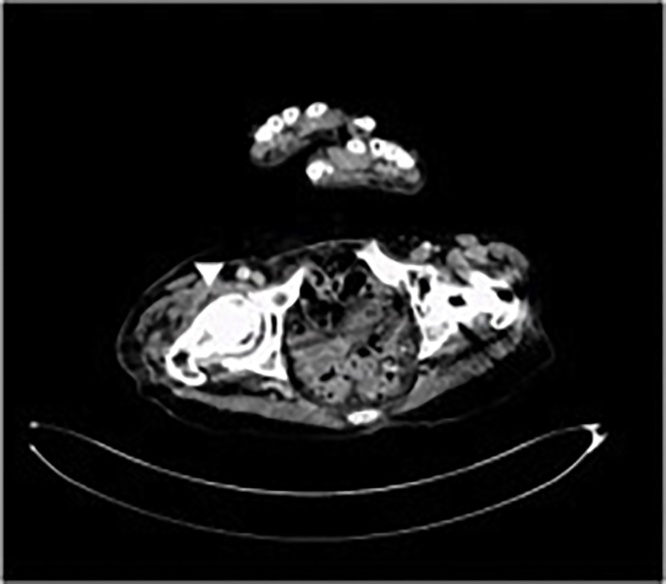
**Contrast-enhanced computer tomography.** The increased joint fluid of right hip (white arrowhead).

An 80-year-old woman presented to our hospital with severe right hip pain that had been worsening for 7 days. Her initial temperature, white blood cell (WBC) count, and C-reactive protein (CRP) were 35.8 °C, 6700/mm^3^, and 34.3 mg/dl, respectively. The initial blood culture revealed the presence of *Micromonas micros.* Radiography revealed osteoarthritis of the right hip joint (Fig. 1-a). Contrast-enhanced computed tomography showed destruction of the right femoral head and increased joint fluid in the right hip (Fig. 2). She was diagnosed as having septic shock associated with acute septic arthritis of the right hip joint. Her medical history included bilateral osteoarthritis of the hip joints, and she underwent left cementless THA (Fig. 1-a). The patient had no relevant drug history, family history (including any relevant genetic information), or psychosocial history. The findings and management plan were discussed with the patient. After obtaining her informed consent, the patient was prepared and shifted for surgery.

The duration from the initial symptom presentation to the 1st-stage operation was 20 days to allow for treatment of septic shock. The duration of antibiotic administration before operation was 20 days (intravenous penicillin and clindamycin). Preformed antibiotic-loaded cement spacers were implanted in the 1st-stage operation. Synovial fluid of the hip joint was obtained for culture. All potentially infected, necrotic, and ischemic tissues were removed. Thereafter, a high-pressure pulsatile lavage with 21 L of lactated Ringer solution was performed. After reaming of the femoral canal, a preformed antibiotic-loaded cement spacer (HIP CEMENT SPACER MOLD, Zimmer Biomet, Warsaw, IN, US) with reinforcement of a 2.4-mm Kirschner wire was implanted (Fig. 1-b). Two grams of vancomycin (Sawai Pharmaceutical Co., Ltd., Osaka, Japan) were added per 40 g Cemex RX (Tecres S.p.A., Italy). The 1st stage operative time required 79 min. The total blood loss in the 1st-stage operation was 340 mL (intraoperative blood loss, 100 mL; postoperative blood loss, 240 mL). In the 1st-stage operation, the culture of the hip joint fluid was negative. The suction drain was removed when there was less than 50 mL clear fluid/24 h. The period of drain insertion was 5 days after the 1st-stage operation. In accordance with the sensitivity profile of the causative organism in the initial blood culture, antibiotics were administered after the 1st-stage operation (intravenous penicillin and clindamycin for 15 days). Oral antibiotics (amoxicillin and clindamycin for 16 days followed by clindamycin for 73 days) were continued until the day before the 2nd-stage operation. The interval to the 2nd-stage operation was 104 days. Weight bearing on the operated extremity was toe-touch weight bearing with crutches. Complications between the 1st- and 2nd-stage operations were caused by drug eruptions attributed to amoxicillin. The WBC count and CRP before the 2nd-stage operation was 4400/mm^3^ and 0.22 mg/dl, respectively.

**Fig. 3:**
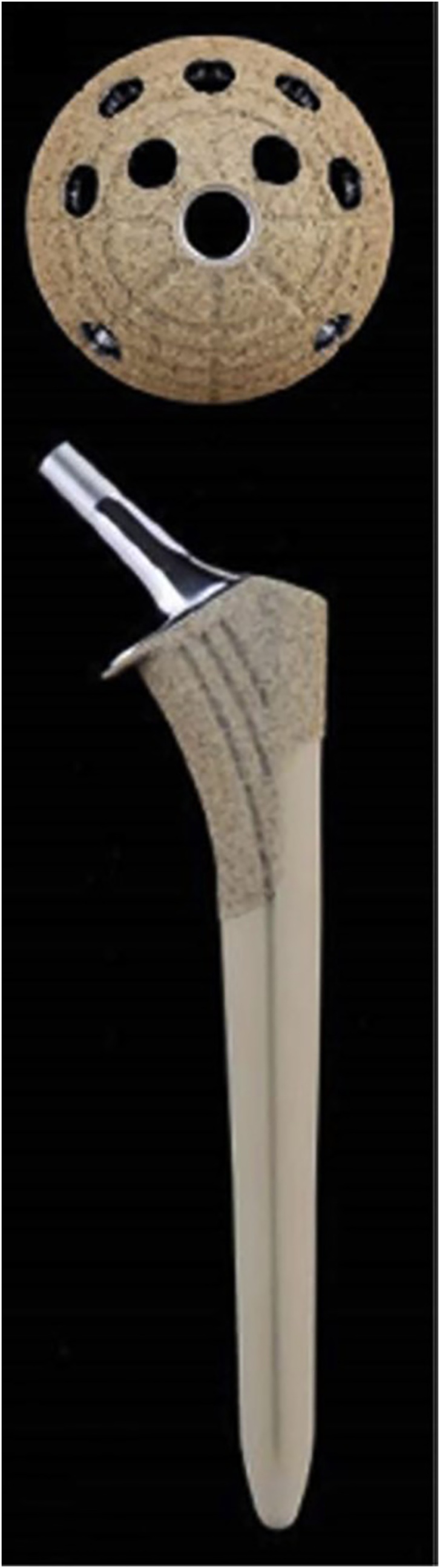
Silver-containing hydroxyapatite coating implant.

In the 2nd-stage operation, a preformed antibiotic-loaded cement spacer was removed, and debridement of the necrotic and ischemic tissue was performed. Synovial fluid was obtained for culture. After a high-pressure pulsatile lavage with 9 L of lactated Ringer solution, the hips were reconstructed using the cementless implants. The acetabular component was AG-PROTEX cup (Kyocera, Tokyo, Japan). The femoral component was AG-PROTEX stem (Kyocera, Tokyo, Japan) (Fig. 3). The operative time for the 2nd stage was 65 min. The total blood loss during the 2nd-stage operation was 550 mL (intraoperative blood loss, 440 mL; postoperative blood loss, 110 mL). In the 2nd-stage operation, cultures of the hip joint fluid were negative. The suction drain was removed 2 days after the 2nd-stage operation as per routine. Antibiotic administration duration after the 2nd-stage operation was 85 days (intravenous cefazolin for 1 day followed by oral clindamycin or 84 days). Walking training was started without limitation of weight-bearing within the allowable pain range beginning 2 days after surgery.

The follow-up duration after the 2nd-stage operation was 28 months. There was no complication or recurrence after 2nd-stage operation. WBC count and CRP at final follow-up were 2600/mm^3^ and 0.03 mg/dl, respectively. Plain radiograph at final follow-up showed no loosening around acetabular and femoral component (Fig. 1-c). The patient was well and showed agreement with the positive results.

The study protocol adhered to the ethical guidelines of the 1975 Declaration of Helsinki, and the study was approved by the institutional review board. The patient was informed that this case study would be submitted for publication, and she provided consent.

## 3 Discussion

SAHJ commonly occurs among children and is relatively uncommon among adults. The incidence in adults is 2–10 per 100,000 person-years [6]. Risk factors of septic arthritis include age, rheumatoid arthritis, diabetes mellitus, and skin infection [7]. The common causative organisms of septic arthritis are *Staphylococcus aureus* and *S. pyogenes* [8].

Arthroscopic debridement is the current standard for early onset of SAHJ without extra-articular abscess formation and advanced osteochondral lesions owing to the lesser degree of invasiveness and shorter hospital stays than are associated with open debridement [1,9]. In progressed SAHJ, both infection control and treatment of destructed joint are needed. Previous studies reported direct THA, Girdlestone arthroplasty, THA after Girdlestone arthroplasty, and two-stage THA with an interval antibiotic-loaded cement spacer for the treatment of SAHJ [2,3,10,11]. Direct THA on SAHJ has a high periprosthetic infection rate of 22% [10]. Girdlestone arthroplasty had a good infection control; however, it was associated with leg length discrepancy, unstable joint, pain, and ultimately poor long-term function [11]. THA after Girdlestone arthroplasty had a periprosthetic infection rate of 8.3% [11]. In previous studies, infection recurrence rates of 0%–13% after two-stage THA have been reported [1,2].

The implant choice for 2nd-stage operation (cementless or cement implant) remains unclear. Previous studies have demonstrated that the use of cementless implants was effective in the 2-stage surgery of SAHJ and periprosthetic THA infections [1]. Recent research also revealed that cementless implant or cement implant with antibiotic-loaded bone cement in the 2-stage surgery of periprosthetic THA infections was not associated with rate of infection recurrence [12].

While a risk of infection recurrence after a two-stage THA remains, recent studies reported the presence of bacteria within the bone tissue in an osteomyelitis model [1,2,13]. Therefore, in addition to a thorough two-stage THA, the Ag-HA coating implant was selected to further reduce the risk of infection recurrence.

A silver-coated mega-prosthesis has been used previously; however, concentrated silver is toxic to osteoblasts, suppresses ossification, and implicates in osteolysis and postoperative prosthesis loosening [[14], [15], [16]]. The maximum amount of silver contained in the Ag-HA implant used in the current study was 2.9 mg, which is considerably lower than that in a silver-coated megaprosthesis [17]. In basic studies, Ag-HA implants were found to have antibacterial activity within the bone, osteoconductive properties, and no adverse reactions *in vivo* [[18], [19], [20]]. Moreover, no adverse events due to silver were reported in a clinical and radiographic study [21]. Besides a thorough two-stage THA, Ag-HA implants may be particularly effective for patients with a high risk of infection, such as our case. However, a larger sample of patients with longer follow-up is required to confirm the safety and efficacy of Ag-HA implants.

## 4 Conclusion

This case demonstrated successful infection control at the 28-month follow-up. To further reduce the infection after the two-staged THA for SAHJ, antibacterial implants, such as Ag-HA implants, may be used; however, long-term studies with additional cases of Ag-HA implants are needed.

## Declaration of competing interest

The authors declare that there is no conflict of interest regarding the publication of this article.

## Funding

This research did not receive any specific grant from funding agencies in either the public, commercial, or not-for-profit sectors.

## Ethical approval

The study protocol adhered to the ethical guidelines of the 1975 Declaration of Helsinki. In addition, the study was approved by the institutional review board of our institute.

## Consent

Written informed consent was obtained from the patient for publication of this case report and accompanying images. A copy of the written consent is available for review by the Editor-in-Chief of this journal on request.

## Author contribution

Akira Hashimoto: Manuscript writing; Original draft preparation

Motoki Sonohata: Corresponding

Masaru Kitajima: Manuscript writing; Review and Editing

Shunsuke Kawano: Manuscript writing; Review and Editing

Shuichi Eto: Manuscript writing; Review and Editing

Masaaki Mawatari: Supervision

## Registration of research studies

This case report is not applicable for registration.

## Guarantor

Motoki Sonohata.

## Provenance and peer review

Not commissioned, externally peer-reviewed.
